# Morphological and Molecular Biological Characteristics of Experimental Rat Glioblastoma Tissue Strains Induced by Different Carcinogenic Chemicals

**DOI:** 10.3390/biomedicines12040713

**Published:** 2024-03-22

**Authors:** Alexandra Sentyabreva, Ekaterina Miroshnichenko, Daria Artemova, Anna Alekseeva, Anna Kosyreva

**Affiliations:** 1 Avtsyn Research Institute of Human Morphology of “Petrovsky National Research Centre of Surgery”, 117418 Moscow, Russia; 2 Research Institute of Molecular and Cellular Medicine, Peoples’ Friendship University of Russia (RUDN University), 117198 Moscow, Russia

**Keywords:** glioblastoma, animal models, tumor tissue strain, high-grade tumor, intratumoral heterogeneity

## Abstract

Glioblastoma (GBM) is a highly aggressive human neoplasm with poor prognosis due to its malignancy and therapy resistance. To evaluate the efficacy of antitumor therapy, cell models are used most widely, but they are not as relevant to human GBMs as tissue models of gliomas, closely corresponding to human GBMs in cell heterogeneity. In this work, we compared three different tissue strains of rat GBM 101.8 (induced by DMBA), GBM 11-9-2, and GBM 14-4-5 (induced by ENU). Materials and methods: We estimated different gene expressions by qPCR-RT and conducted Western blotting and histological and morphometric analysis of three different tissue strains of rat GBM. Results: GBM 101.8 was characterized by the shortest period of tumor growth and the greatest number of necroses and mitoses; overexpression of *Abcb1*, *Sox2*, *Cdkn2a*, *Cyclin D*, and *Trp53*; and downregulated expression of *Vegfa*, *Pdgfra*, and *Pten*; as well as a high level of HIF-1α protein content. GBM 11-9-2 and GBM 14-4-5 were relevant to low-grade gliomas and characterized by downregulated *Mgmt* expression; furthermore, a low content of CD133 protein was found in GBM 11-9-2. Conclusions: GBM 101.8 is a reliable model for further investigation due to its similarity to high-grade human GBMs, while GBM 11-9-2 and GBM 14-4-5 correspond to Grade 2–3 gliomas.

## 1. Introduction

Glioblastoma (GBM), formerly known as glioblastoma multiforme, is the most aggressive diffuse glioma and the most common brain and CNS malignancy, accounting for almost a half of all malignant primary brain and CNS tumor cases. It has a rather poor prognosis, with a median survival of 7.9–15 months post-diagnosis [1,2], whereas only 1.5–5% of patients survive 5 years post-diagnosis [1,2]. “Multiforme” stood for the extreme variability, including intratumoral, of this type of neoplasm, which is the cause of its high malignancy and predominant therapy resistance.

The fifth and latest edition of the World Health Organization (WHO) classification of tumors of the human central and peripheral nervous system (WHO CNS5) is now based on the most recently discovered prognostically important molecular genetic features over morphological hallmarks [3,4]. One of the most significant changes in WHO CNS5 is the subdividing of high-grade diffuse gliomas into isocitrate dehydrogenase (IDH) wild and IDH mutant types. GBM IDH wildtype is prevalent (~90% of all cases), mostly primary, and always regarded as Grade 4; whereas the GBM (or astrocytoma, according to WHO CNS5) IDH mutant is frequently characterized as Grade 2 or 3, and Grade 4 is a more common feature of the secondary one and so is considered as more prognostically optimistic [5]. At the same time, another pivotal characteristic of the GBM IDH wild type is O6-methylguanine-DNA methyltransferase (MGMT) promoter methylation. It occurs in 30–40% of all GBM cases and potentially correlates with a higher efficacy of temozolomide-based therapy [4,6]. However, this aspect must only be taken into account among other markers of high-grade malignancy and shortened overall survival predictors, such as age, ki-67 ≥ 30%, alterations in p53, cyclin-dependent kinases CDK4/6, phosphatase and tensin homolog (PTEN), fibroblast growth factor receptor 2, and some others [4,7,8].

Despite increased knowledge and understanding of the molecular evolution of GBM, it remains a scientific and medical challenge as an incurable disease with extremely poor survival. For now, there are various GBM cell lines of rat and mouse origin. The most widely used and studied rat models are chemically induced C6 glioma, 9L gliosarcoma, and F98 glioma; GL261 and CT-2A represent mouse models of chemically induced GBMs; also, some xenografts of patient-derived and genetically engineered mouse models were developed recently. However, all these models, even C6, possess some critical disadvantages: moderate/high immunogenicity, while human high-grade GBMs are non-immunogenic; pronounced genetic distinctions, as well as a lack of intratumoral heterogeneity; variability in tumor formation and among line samples; slow or inconsistent growth; or requiring immunocompromised rodents [9,10]. Thus, relevant, accessible, and accurately reproducible models are still essentially needed for peer and diversified investigation of GBM biology, including the signaling cascades and mechanisms involved in initiation, survival, unrestricted proliferation, neovascularization, metastasis, and resistance to different treatment approaches.

The collection of experimental tumors of the nervous system and neural tumor cell lines was formed in the 1970s at the neuromorphology laboratory of the Avtsyn Research Institute of Human Morphology. It contains tumor cell lines and transplanted tissue strains of tumors of the nervous system of humans, rats, mice, and rabbits. Cell lines consist of one type of immortalized cell, so there is no heterogeneity among them. Unlike cell lines, tissue strains contain different cell types, including tumorigenic ones, at various differentiation stages, as well as normal ones such as astrocytes and oligodendrocytes. Tissue strains cannot be cultivated in vitro, they must be inoculated in an animal to reproduce. It is more difficult and expensive to work with tumor tissue strains, but they mimic human glioma heterogeneity considerably more closely than any cell line or combination of several ones. In particular, there are various experimental models of diffuse high-grade gliomas: rat GBM 101.8, 11-9-2, and 14-4-5 [11,12]. The GBM 101.8 strain was originally obtained by an intracranial placement of a bolus containing 1 mg of 7,12-Dimethylbenz[a]anthracene (DMBA). It is a particularly aggressive strain, which was used for investigation of chemotherapy for GBM and could be a potentially highly reliable model of human high-grade glioma [13,14,15]. Both GBM 14-4-5 and GBM 11-9-2 strains were obtained from the offspring of rats that were administered an intravenous injection of 75 µg of N-ethyl-N-nitrosourea (ENU) performed on the 20th day of pregnancy [11,12]. The grown tumors were dissected, stabilized, and multiplied by several passages from one rat to another, and then stored at −70 °C until the next revitalization was needed. DMBA- and ENU-induced neoplasms are considered quite relevant models for studying tumors of different localizations, since these chemicals trigger processes that accurately correspond with complex and multi-stage ones in de novo carcinogenesis [16,17,18]. It is worth noting that the same exposure could lead to the development of a neoplasm of the same localization but possessing different features. Hence, the purpose of this work is establishing morphological and molecular biological characteristics of three GBMs to reveal their similarities and distinctions, as well as determine their level of correspondence to human GBMs. The work was carried out on the basis of a unique scientific facility “The Collection of experimental tumors of the nervous system and neural tumor cell lines” lead by Kosyreva A.M. (https://med.ru/ru/unu-kollekcia-eksperimentalnyh-opuholei-nervnoi-sistemy-i-neiralnyh-opuholevyh-kletocnyh-linii, (accessed on 31 January 2024)).

## 2. Materials and Methods

### 2.1. Animals

The experiment was performed on adult male Wistar rats (*n* = 27, 3 months old, weight 220–250 g), approved by the Bioethics Committee of the Avtsyn Research Institute of Human Morphology (Protocol No. 29 (5), 8 November 2021). Animals were kept in plastic cages (60 × 38 × 18.5 cm) in social groups of 4–5 animals each with free access to food and water. The temperature in the vivarium room was maintained within 18–22 °C, and the air humidity was 50–65%. All experimental work was performed according to Directive 2010/63/EU of the European Parliament and of the Council of the EU on the protection of animals used for scientific purposes (Strasbourg, 22 September 2010).

### 2.2. Tumor Inoculation

The intracranial inoculation of GBM 14-4-5, GBM 11-9-2, and GBM 101.8 (~10^6^ cells) was performed on 18 animals (*n* = 6 per strain) as described previously [19]. The animals were anesthetized with 100 mg/kg ketamine and 10 mg/kg xylazine intraperitoneally. Parietal skin surface was treated with antiseptics and a 10 mm-length longitudinal incision was made. Tumor transplantation into the brain was performed sterilely through a burr hole, applied with a dental spherical burr 1.5 mm in diameter, in the parietal bone of the skull. The inoculation point located at a distance of 2 mm to the right of the sagittal suture (*Sutura sagittalis*) and 2 mm caudally from the coronal suture (*Sutura coronalis*). The crushed tumor tissue was implanted with a trocar to a depth of 4 mm in the area of the striatum. After replanting the tumor, the wound was treated with sulfanilamide, and the skin wound were sutured with vicryl thread. According to the literature, GBMs manifest clinically with the loss of animals’ body weight when the terminal stage of tumor growth is reached [20,21]. The animals were weighted just before tumor inoculation on Day 0, then on post-inoculation Day 10, and daily afterwards. The body weight loss began on post-inoculation Day 14–18 in animals with GBM 101.8, as previously described [21], on post-inoculation Day 25–29 in animals with GBM 11-9-2, and on post-inoculation Day 33–37 in animals with GBM 14-4-5.

### 2.3. Samples Obtaining and Histological Preparations

Animals were euthanized by overdose (15 mg/kg) of Tiletamine + Zolazepam (Zoletil, Vibrac Sante Animale, France). The whole brains were fixed in 10% buffered formalin (BioVitrum, Saint Petersburg, Russia) for 48 h, then dehydrated with ethanol of ascending concentration, cleared with xylene, and paraffin-embedded by a standard procedure. Serial 4–5 μm-thick histological sections were stained with hematoxylin and eosin (BioVitrum, Saint Petersburg, Russia).

### 2.4. Morphology and Morphometry

Histological examination of tumors was randomized and blinded. Necrotic lesions were measured in a semi-quantitative way over the entire tumor area in a section using ImageScopeM software (version 12.3) interactively with a Leica DFC290 camera (Leica Microsystems, Wetzlar, Germany). Mitosis numbers were counted over the entire tumor area and calculated as the mean number of mitotic figures per field of view, at magnification ×400.

### 2.5. Quantitative Real-Time Polymerase Chain Reaction (qPCR-RT) Evaluation of Gene Expression

The mRNA expression was assayed by real-time qPCR in tissue fragments of the tumors, preserved in IntactRNA solution (Eurogen, Moscow, Russia) and stored on −20 °C until being studied. The performed analysis included the detection and evaluation of expression levels of stem cell markers (*Cd133* and *Sox2*), cell cycle regulatory markers (*Cdkn2a*, *Cyclin D*, *Trp53*, and *Pten)*, markers of neovascularization and adaptation to hypoxia (*Hif-1α*, *Vegfa*, and *Pdgfra*), and chemotherapy resistance markers (*Mgmt* and *Abcb1a*). These particular markers are used to establish the GBM malignancy grade, as well as to predict the probable resistance to various therapy approaches and overall prognosis. The levels of all followed mRNA expression relative to the gene glyceraldehyde 3-phosphate dehydrogenase (*Gapdh*) expression level as a reference [22] were determined using qPCRmix-HS SYBR (Eurogen, Russia) with the fluorescent intercalating dye SYBR Green I. Amplification, detection, and digital analysis of fluorescence levels in real time was performed on a DT-96 Real-Time PCR Cycler (DNA-Technology JSC, Moscow, Russia) in a standard mode at 95 °C for 5 min followed by 95 °C for 15 s, 62 °C for 10 s + reading, and 72 °C for 20 s ×45. Obtained data were analyzed as described before [23]. All the primers’ sequences were selected precisely for rat species by online soft Primer-BLAST (Table A1).

### 2.6. Western-Blotting (WB) Analysis

Tissue was lysed in protein solubilization buffer (PSB) from MicroRotofor™ Cell Lysis Kit (Mammal) (#1632141, Biorad, Hercules, CA, USA) and centrifuged at 11,500× *g* and +4 °C for 30 min. Then, the supernatant was mixed in a proportion of 1:1 with 2× Laemmli Sample Buffer c β-mercaptoethanol, and heated for 5 min at +65 °C. Protein separation was carried out in 4–15% sodium dodecyl sulfate–polyacrylamide gel electrophoresis (SDS-PAGE). After electrophoresis, the proteins from the gel were transferred to a polyvinylidene difluoride (PVDF) membrane in the Trans-Blot Turbo Transfer System (Biorad, CA, USA) at 25 V, 1 A for 45 min. After transfer, the membranes were washed with deionized water and nonspecific binding sites were blocked using a 5% solution of Blotting-Grade Blocker (#1706404, Biorad, CA, USA) at room temperature for 1 h. Then, the membranes were incubated for 24 h at +4 °C in a solution of primary antibodies: rabbit anti-HIF-1a (PAA798Ra01, Cloud-Clone Corp., Houston, TX, USA 1:500), rabbit anti-CD133 (ab19898, Abcam, Boston, MA, USA, 1:2000) and mouse anti-GAPDH (Cat. No. 5G4, clone 4G5, HyTest, Moscow, Russia) (1:5000). After incubation with primary antibodies, membranes were washed in TBST and incubated at room temperature for 1 h in a solution of secondary antibodies: goat-anti-rabbit IgG-HRP (ab6721, Abcam, 1:5000) and goat-anti-mouse IgG-HRP (ab6789, Abcam, 1:2000). After incubation with secondary antibodies, membranes were washed in TBST and developed using the Clarity Western ECL Substrate (#1705060, Biorad, Hercules, CA, USA). The chemiluminescence signal was detected on a ChemiDoc Imaging System (Biorad, CA, USA) and Image Lab Touch Software (version 3.0.1) (Biorad, Hercules, CA, USA). The GAPDH protein was used as a reference protein for application; the signals of target proteins were normalized to the signal level of this protein, then target proteins’ levels were evaluated by adjusted volume relative to one of the reference protein.

### 2.7. Statistics

The statistical analysis was performed using GraphPad Prism 8.0.1 (Boston, MA, USA) via nonparametric and multiple comparison procedures. Multiple comparisons used the Kruskal–Wallis test followed by Dunn’s post hoc tests. Data are displayed as box-and-whisker plots of median, upper–lower quartile, and upper–lower extreme values with all data points shown. Differences were considered significant at *p*  < 0.05.

## 3. Results

### 3.1. General Morphological Characteristics

**Figure 1:**
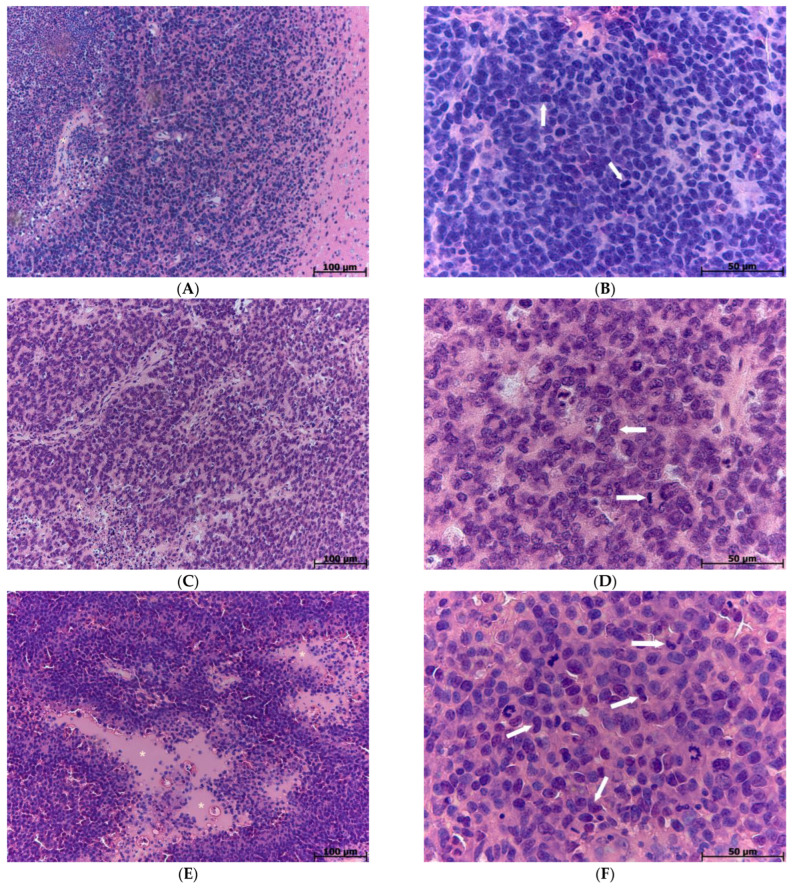
Necrosis areas ((**A**,**C**,**E**); indicated as *) and mitoses ((**B**,**D**,**F**); indicated as ↑) in GBM 14-4-5, GBM 11-9-2, and GBM 101.8. Hematoxylin and eosin staining, magnification ×200, ×640.

Tumors of GBM 14-4-5 tissue strain were characterized by small polymorphonuclear cells of a monomorphic shape. Single mitoses were detected per the field of view. There were a small number of partially formed vessels and large necroses at the border of the tumor and healthy brain tissues (Figure 1A,B).

Tumors of GBM 11-9-2 tissue strain contained a high number of polymorphonuclear cells of a polymorphic shape. A moderate amount of mitoses was observed, as well as extensive necrosis areas filled with tissue detritus and deteriorated nuclei fragments. The tumors had a large number of partially formed vessels and endothelial cell proliferates (Figure 1C,D).

Tumors of GBM 101.8 tissue strain characterized by an infiltrative type of growth, and tumor cells were diffusely scattered in the peritumoral zone and formed several clusters of glandular structures around neurons. Tumor cells were highly polymorphic in shape and size with hyperchromic nuclei and a narrow, almost indistinguishable rim of the cytoplasm. Multiple foci of necrosis were observed, including ones surrounded by perpendicularly located elongated tumor cells, so-called palisade-shaped necroses, which is one of the histological criteria of human high-grade GBM. There were a significant number of mitoses and of dying cells as well. In all the tumors of this type, there were a high number of poorly formed vessels with uneven wide rounded lumens. Reticulate edema was observed in the peritumoral zone, and neurons nearby the border were predominately hyperchromic (Figure 1E,F).

A semi-quantitative analysis of the necrosis area and mitoses numbers is summarized in Table 1.

### 3.2. qPCR-RT Examination of Tumor Tissues

#### 3.2.1. Stem Cells Markers

**Figure 2:**
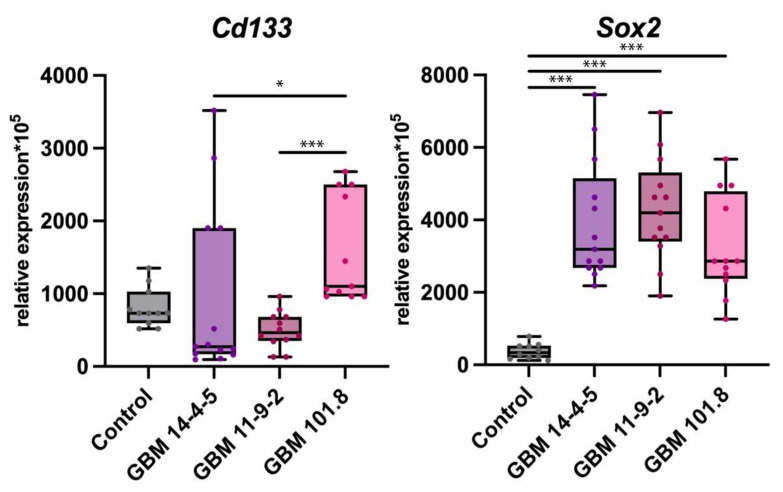
mRNA expression levels of *Cd133* and *Sox2* in the intact brain tissue of healthy control rats (*n* = 6), GBM 14-4-5 (*n* = 6), GBM 11-9-2 (*n* = 6), and GBM 101.8 (*n* = 6). *—*p* < 0.05, ***—*p* < 0.001. The data are displayed as a line—median, box—25–75 quartiles, whiskers—non-outlier range. The Kruskal–Wallis test was used for multiple comparisons.

In the results of qPCR-RT of the tumors’ tissue fragments, no difference in *Cd133* expression was detected among all groups relative to the one obtained from the intact brain tissue as a control. However, it was significantly higher in GBM 101.8 compared with both GBM 14-4-5 and GBM 11-9-2. At the same time, the level of *Sox2* expression was dramatically upregulated in all three GBM strains compared to the control, whereas no difference in it was observed among different tumor strains (Figure 2).

#### 3.2.2. Cell Cycle Regulatory Markers

**Figure 3:**
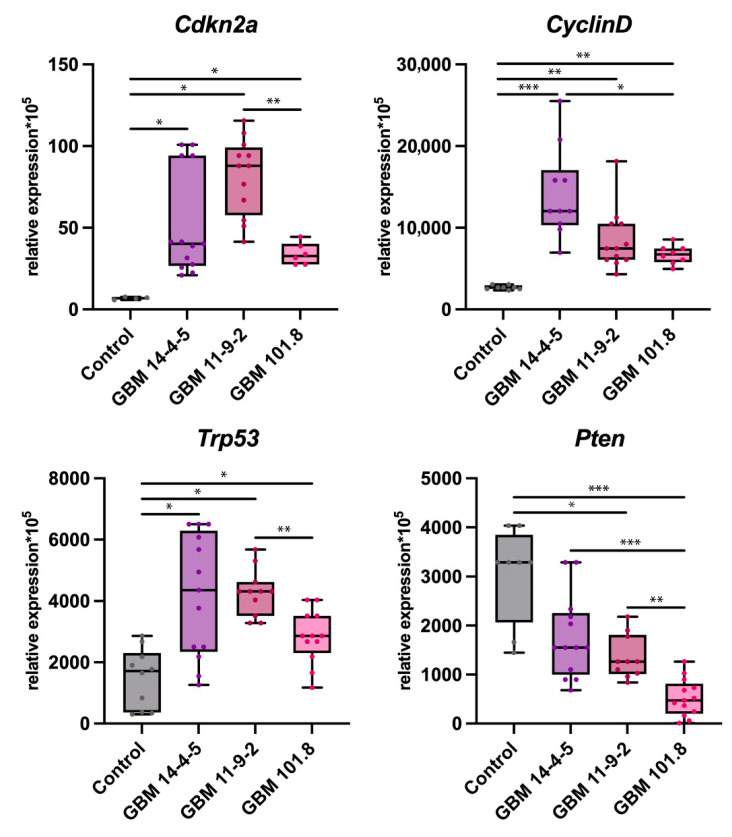
mRNA expression levels of *Cdkn2a*, *Cyclin D*, *Trp53*, and *Pten* in the intact brain tissue of healthy control rats (*n* = 6), GBM 14-4-5 (*n* = 6), GBM 11-9-2 (*n* = 6), and GBM 101.8 (*n* = 6). *—*p* < 0.05, **—*p* < 0.005, ***—*p* < 0.001. The data are displayed as a line—median, box—25–75 quartiles, whiskers—non-outlier range. The Kruskal–Wallis test was used for multiple comparisons.

The level of cyclin-dependent kinase inhibitor 2A (*Cdkn2a*) expression, which is one of the key suppressors of cell cycle progression, was notably upregulated in all three GBMs relative to intact brain tissue. However, in GBM 101.8 it was substantially lower than in GBM 11-9-2. The same tendency was observed for the expression level of *Cyclin D*, which is the main target for Cdkn2a to bind. The *Trp53* gene encodes the p53 protein, which is one of the most potent oncosupressors. Its expression was also markedly higher in all three tumors compared with the control, as well as in GBM 11-9-2 compared with GBM 101.8. While *Cdkn2a* and *Trp53* usually rise in tumor cells unless mutated, in malignant neoplasms, there is commonly observed a severe downregulation of the phosphatase and tensin homolog deleted on chromosome 10 (*Pten)*, which is one of the key suppressors of the pro-oncogenic PI3K/AKT/mTOR pathway and a pivotal marker of tumor malignancy grade. Its levels of expression were considerably lower in GBM 11-9-2 and GBM 101.8 compared to the healthy control, as well as in GBM 101.8 where it was downregulated more pronouncedly compared with both GBM 14-4-5 and GBM 11-9-2 (Figure 3).

#### 3.2.3. Adaptation to Hypoxia and Neovascularization Markers

**Figure 4:**
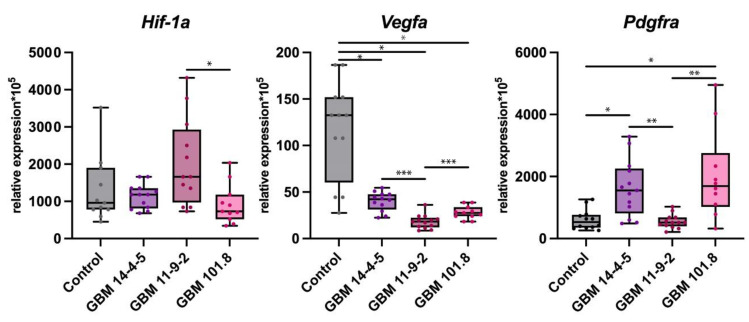
mRNA expression levels of *Hif-1α*, *Vegfa*, and *Pdgfrα* in the intact brain tissue of healthy control rats (*n* = 6), GBM 14-4-5 (*n* = 6), GBM 11-9-2 (*n* = 6), and GBM 101.8 (*n* = 6). *—*p* < 0.05, **—*p* < 0.005, ***—*p* < 0.001. The data are displayed as a line—median, box—25–75 quartiles, whiskers—non-outlier range. The Kruskal–Wallis test was used for multiple comparisons.

Hypoxia-inducible factor 1 alpha (*Hif-1α*) is a key transcription factor initiating the very first line of cell response to hypoxia and launching multiple downstream events including intratumoral angiogenesis. Vascular Endothelial Growth Factor A (*Vegfa*) and Platelet Derived Growth Factor Receptor Alpha (*Pdgfrα*) are responsible for neovascularization and promoted by the increase in HIF-1α. No notable upregulation was detected in all three GBMs relative to intact brain tissue, although the *Hif-1α* expression level was significantly higher in GBM 11-9-2 than in GBM 101.8. The expression level of *Vegfa* was considerably downregulated in all GBM 14-4-5, GBM 11-9-2, and GBM 101.8. However, it was downregulated considerably in GBM 11-9-2 in comparison with GBM 14-4-5 and GBM 101.8. At the same time, *Pdgfrα* expression was upregulated markedly in GBM 14-4-5 and GBM 101.8 relative to intact brain tissue. It was also significantly higher in these two strains compared with GBM 11-9-2 (Figure 4).

#### 3.2.4. Multiple Drug Resistance Marker

**Figure 5:**
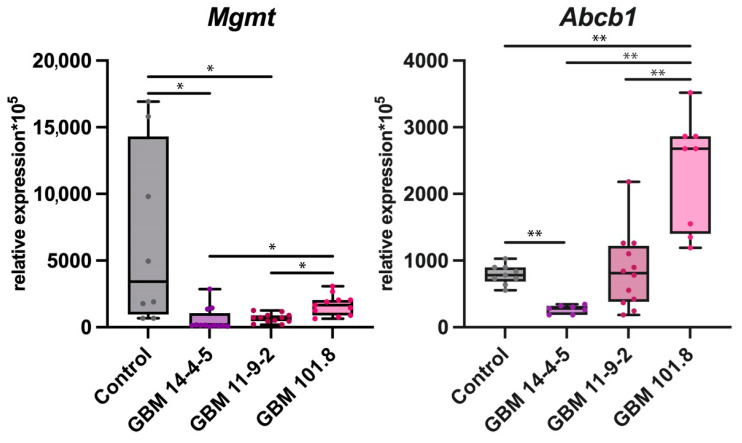
mRNA expression levels of *Mgmt* and *Abcb1* in the intact brain tissue of healthy control rats (*n* = 6), GBM 14-4-5 (*n* = 6), GBM 11-9-2 (*n* = 6), and GBM 101.8 (*n* = 6). *—*p* < 0.05, **—*p* < 0.005. The data are displayed as a line—median, box—25–75 quartiles, whiskers—non-outlier range. The Kruskal–Wallis test was used for multiple comparisons.

MGMT is a member of the DNA repair enzymes large family. This particular enzyme enhances the resistance of tumor cells to alkylation-induced damage and, as a result, to alkylating agents-based chemotherapy drugs, such as temozolomide. The *Mgmt* mRNA expression level was considerably downregulated in GBM 14-4-5 and GBM 11-9-2 but not in GBM 101.8 relative to the control, as well as in both GBM 14-4-5 and GBM 11-9-2 compared with GBM 101.8. Another pivotal marker of the potential chemotherapy resistance of GBMs is the ATP binding cassette subfamily B member 1 (*Abcb1*), which is capable of maintaining the drug molecules’ efflux. The level of *Abcb1* expression was the same in GBM 11-9-2, downregulated in GBM 14-4-5, and upregulated in GBM 101.8 relative to the healthy control. It was also significantly higher in GBM 101.8 than in both GBM 14-4-5 and GBM 11-9-2 (Figure 5).

### 3.3. Western Blotting Analysis of Proteins’ Content in Tumor Tissues

**Figure 6:**
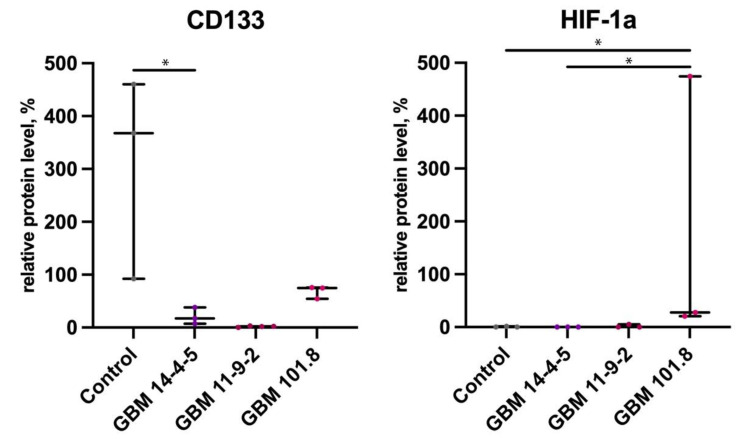
Relative protein levels of CD133 and HIF-1a in the intact brain tissue of healthy control rats (*n* = 3), GBM 14-4-5 (*n* = 3), GBM 11-9-2 (*n* = 3), and GBM 101.8 (*n* = 3) tumor strains. *—*p* < 0.05. The data are displayed as a line—median, box—25–75 quartiles, whiskers—non-outlier range. The Kruskal–Wallis test was used for multiple comparisons.

Additionally, WB analysis was performed to evaluate the content of some proteins in tumors’ tissues. The CD133 protein content decreased in GBM 11-9-2 relative to the healthy control and GBM 101.8. At the same time, the content of HIF-1α was significantly higher in GBM 101.8 relative to the healthy control and GBM 11-9-2 but not GBM 14-4-5 (Figure 6).

## 4. Discussion

In our work, we examined three different GBM tissue strains, which were originally induced by two different carcinogenic chemicals. An intracranial injection of DMBA has led to development of the GBM 101.8 strain, whereas an intravenous injection of ENU performed on pregnant rats has caused the development of both GBM 14-4-5 and GBM 11-9-2 in their offspring. Just as in the initiation and growth of similar tumors, gliomas in particular can occur due to various pro-carcinogenic effects, as the same carcinogens might cause the development of neoplasms with distinct feature sets, which would predispose different responses to the same therapeutic approaches. The profound phenotypic analysis of molecular biological features of these strains will allow us to determine their similarities and distinctions, as well as the level of correspondence to human GBM grades [3] and suitability for further research.

Human glioblastomas are extremely heterogeneous tumors. GBM heterogeneity and the response to treatment are determined not only by intrinsic factors, but also vary greatly between new and recurrent GBM, as well as in case of the treatment attempt of naïve or therapy-experienced patients [24]. GBMs have been classified into four subtypes: classical, mesenchymal, proneural, and neural. These subtypes are mainly distinguished by the expression levels of EGFR, NF1, and PDGFRA/IDH1 and predispose to different sensitivity to various treatment approaches [1]. However, according to single-cell sequencing data, there are differences in the gene expression profiles even within these subtypes [25]. Moreover, differences in the expression profile of genes such as EGFR, PTEN, and PDGFR were observed within the same tumor nodi, which apparently indicate several cell lineages coexisting in the same tumors [26]. Therefore, it is quite challenging to compare the tissue models described in our work with human GBMs. However, many promising similarities were discovered.

The morphofunctional and molecular biological characteristics of examined GBMs are summarized in Table 2.

According to the histological characteristics of tissue tumor strains, these models are considerably similar to high-grade human glioblastomas in many respects, such as the presence of pseudopalisading and landscape-like necrosis areas, a high number of mitoses and apoptotic dying cells, and the presence of endothelial rosettes and newly formed vessels. Although the WHO CNS5 guidelines indicate the importance of molecular biological features to establish the GBM grade more accurately [3,4], these morphological characteristics are still relevant. Despite the growth and consequent critical hypoxia inside the tumoral nodi being inevitable, as well as imperfect vasculogenesis and necrosis areas emerging, in GBM 14-4-5 and GBM 11-9-2 these morphological features were substantially less vigorous in comparison with GBM 101.8. Their growth periods were also longer by 2.2 times (35 vs. 16 days of GBM 101.8) and 1.7 times (27 vs. 16 days of GBM 101.8), correspondingly.

Stem cell markers are among the most essential ones for tumorigenesis and growth, as well as for the prognosis of patients, since their presence determines therapeutic approaches and decisions. Currently, CD133 is considered as one of the most potent and significant cancer stem cells (CSCs) markers, potentially indicating a poor overall prognosis when increased [27]. Its level of mRNA expression remained unchanged in all GBM 101.8, GBM 14-4-5, and GBM 11-9-2 compared with the healthy control, although the WB data demonstrated a CD133 protein decrease in GBM 11-9-2 relative to the control. However, there was a statistical difference between GBM 101.8 and both GBM 14-4-5 and GBM 11-9-2, where *Cd133* expression was substantially higher. It could occur, among other reasons, due to an increase in HIF-1a protein content [28]. Despite some controversial data in the literature, concerning the high heterogeneity of its distribution and expression in both normal tissues and tumors [29], its upregulated expression and increased protein content strongly correlates with human GBM Grade 4 with lower overall survival [30]. It makes CD133 a promising marker for further study. However, GBMs are one of the most aggressive and radio-/chemotherapy resistant tumors because of their prominent intratumoral heterogeneity, concerning CSC populations as well [27,31]. Another pivotal CSC marker is SOX2, which mRNA expression was upregulated in all three GBM 14-4-5, GBM 11-9-2, and GBM 101.8. It is vital for the progress of numerous physiological developmental processes, whereas its dysregulation might lead to tumorigenesis. SOX2 is responsible for CSCs’ proliferation and differentiation, epithelial-to-mesenchymal transition (EMT), migration, invasion, and metastasis, as well as resistance to apoptosis and different therapy schemes [32,33]. *Sox2* overexpression and the malignancy grade of GBMs correlate positively [34,35], especially when its site is hypomethylated [36]. In a recent study, Lopez-Bertoni et al. discovered its capability of epigenetic suppression of miR-296-5p, which abrogates complex downstream processes of cells’ self-renewal [37]. All these data confirm the importance of SOX2’s presence in tumor strains to perform further investigations concerning the self-maintaining abilities of tumors and overcoming their therapeutic resistance.

One of cancer cell hallmarks is cell cycle disturbances leading to their uncontrolled proliferation. We observed an upregulation of *Cdkn2a* and *Trp53* (*p53*), which are among the most potent onco-suppressors, in each of GBM 14-4-5, GBM 11-9-2, and GBM 101.8 relative to the healthy control. At the same time, the expression level of *Pten*, a highly important suppressor of the pro-oncogenic PI3K/AKT/mTOR signaling network [38], was significantly downregulated in GBM 101.8 and GBM 11-9-2 but not in GBM 14-4-5. The *Cdkn2a* gene encodes the p16 and p14^arf^ proteins responsible for cell cycle control. An increase in Cdkn2a promotes translation of the p16 protein, which inhibits interactions between cyclin D and cyclin-dependent kinases CDK4/6. It causes downstream events leading to cell cycle arrest in the late G1 phase [39]. The p14^arf^ protein is capable of binding the p53-specific E3 ubiquitin ligase Mouse double minute 2 homolog (Mdm2). It prevents their interaction and Mdm2-mediated degradation of p53, so it accumulates and performs it anti-oncogenic functions [40,41,42]. At the same time, PTEN prevents the dephosphorylation of class I phosphoinositide 3 kinase (PI3K) and all PI3K-mediating downstream events, such as enhancing proliferation and glucose metabolism, motility, and genomic instability [38,43]. The PI3K/AKT/mTOR pathway is also involved in cancer-promoting features such as angiogenesis and inflammatory cell recruitment [44]. Polymorphisms and genetic aberrations in the *Cdkn2a*, *Trp53*, and *Pten* genes strongly correlate with the higher malignancy of GBM and a poor overall prognosis, as well as the development of some other types of cancer [38,45]. However, they could remain unmutated, but yet promoted or suppressed in other ways, including epigenetic ones. Sun et al. reported in arecent study that unmutated Trp53 transactivation could be prevented epigenetically by bromodomain-containing protein 8 [46]. It might imply that rather moderate upregulation of *Cdkn2a*, *Cyclin D*, and *p53* expression in GBM 101.8 relative to GBM 14-4-5 and GBM 11-9-2 could have happened due to this or some other epigenetic event, which seems treacherous and worthy of further investigation.

As tumors continue to grow, the higher number and division rate of cancer cells, the more energy supplies they require. Hypoxia is a common event influencing tumor tissue and its microenvironment, since there the imbalance between oxygen delivery and its consumption by cancer calls is inevitable. Both acute and chronic hypoxia eventually lead to an increase in DNA damage and a decrease in its repair, as a result of which tumors might acquire even more aggressive properties and malignant phenotypes, if survived and adapted [47,48]. The main transcription master-regulator managing cells’ adaptation to hypoxic conditions is the HIF-1α protein. It has pronounced pleiotropic effects and is capable of glucose metabolism management [49] and angiogenesis promotion via *Vegfa* and *Pdgfra* expression upregulation [50,51,52]. High levels of HIF-1α in GBMs have been associated with a poor prognosis and low overall survival [53]. In a recent study, our colleagues reported high mortality in Wistar rats susceptible to hypoxia due to the progression of GBM 101.8 and the larger size of tumors and necrosis areas in rats tolerant to hypoxia [14]. Although we did not observe an upregulation of *Hif-1α* in any of the three GBM 101.8, GBM 14-4-5, and GBM 11-9-2, the HIF-1α protein content was increased in GBM 101.8 according to WB data. This could happen due to its accumulation caused by inhibited protein degradation, which is a common event under hypoxic conditions, confirming the high malignancy grade of this strain. The upregulation of *Pdgfra* expression, observed in GBM 101.8 and GBM 14-4-5, also might be considered as a marker of tumor aggressiveness. The PDGFs/PDGFRs network is a potent promoter of proliferation for cells of mesenchymal origin [54], and EMT is a feature of GBMs, including the fact that it might be additionally stimulated by a SOX2 increase. The activation or upregulation of *Pdgf* or *Pdgfra* also may lead to an uncontrolled blood vessel formation in tumors self-maintaining their survival and growth [55], making this pathway a promising treatment target.

Another challenge in GBM therapy is its frequently observed and highly variable multiple drug resistance. The most recent and profound investigations provided essential data concerning GBMs’ genetic and molecular profiles and helped to improve WHO CNS5 by the addition of MGMT promoter methylation, occurring in 30–40% of all cases, or unmethylation as a potent prognostic marker [56]. We observed the pronounced downregulation of *Mgmt* in GBM 14-4-5 and GBM 11-9-2 but not in GBM 101.8. Since the MGMT promoter is methylated, its transcription and translation are abrupted, and the enzyme ceases to exist in such cells. The loss of a key DNA repair enzyme, protecting from alkylation-induced damage caused by chemotherapy drugs, such as temozolomide, makes GBMs more vulnerable to it and improves the overall prognosis. It is worth noting that MGMT’s capability is not limited by only fixing alkylation-induced damage. Radiotherapy alone or combined with temozolomide also becomes more effective [57]. Downregulation of the *Mgmt* expression level in GBM 14-4-5 and GBM 11-9-2 indirectly confirms its promoter methylation and the enzyme silencing, as well as the unchanged *Mgmt* expression level in GBM 101.8 implying its unmethylated state in the same way. Regarding these data, high *Mgmt* expression indicates its potential high therapy resistance in general and temozolomide-based therapy resistance in particular.

Another important marker with a rather different mechanism of action is ABCB1, also known as MDR1 or P-glycoprotein [58]. We observed its upregulation in GBM 101.8. Genomic instability, which is one of the main high-grade GBM features, increases *Abcb1* expression, but it also could be regulated by epigenetic mechanisms [59,60]. It is an ATP-binding cassette transporter functioning as an efflux pump, so it disrupts the intracellular accumulation of various anti-cancer drugs [61]. As an ATP-dependent process, it requires a high intensity of glucose metabolism in tumor cells, and alongside the previously described upregulation of *Hif-1α* and *Pdgfra* its expression increasing is evidence of the potentially high chemotherapy resistance of GBM 101.8.

Our study has certain limitations. The tumors samples were obtained in the late or terminal stage of GBM progression with massive necrosis areas, which might explain the unchanged or downregulated expression of some markers, especially in GBM 101.8. However, this study enlightens the directions of further investigations, including obtaining samples at earlier times and more profound examination of the cross-talk of intra- and peritumoral processes during tumors’ growth.

## 5. Conclusions

Regarding all reported data, tissue tumor strains correspond more closely to human GBMs than cell cultures. Here, we described three tissue strains that differ formidably in growth rate, morphological characteristics, and levels of gene and protein expression. Due to these data, it was observed that GBM 101.8 is a significantly more aggressive tumor than GBM 14-4-5 and GBM 11-9-2, although all three tissue strains displayed heterogeneity of different levels. Hence, these models can be used in future investigations of different mechanisms of different grades of GBMs’ rates progression, as well as in preclinical studies to search for treatment approaches and precise therapy diagnostic algorithms.

## Figures and Tables

**Table 1:** Comparison of necrosis area and mitoses numbers in GBM 14-4-5 (*n* = 6), GBM 11-9-2 (*n* = 6), and GBM 101.8 (*n* = 6) (displayed as Me (25–75%)).

Morphological Feature	GBM 14-4-5	GBM 11-9-2	GBM 101.8
Necrosis areas	++	++	+++
Mean mitosis number per field of view (25,000 µm^2^)	3.0 (2.3–4.1) *	4.7 (2.4–6.5)	6.65 (5.0–8.1) *

++—moderate number of necrosis areas; +++—high number of necrosis areas; * *p* < 0.005, GBM 14-4-5 vs. GBM 101.8.

**Table 2:** Comparative characteristics of GBM 14-4-5, GBM 11-9-2, and GBM 101.8 morphological and genetic molecular phenotypes.

Marker	GBM 14-4-5	GBM 11-9-2	GBM 101.8
General characteristics			
Mean period of tumor growth, days	35	27	16
Necrosis areas	++	++	+++
Mean mitoses number per a field of view (25,000 µm^2^)	+	++	+++
mRNA expression			
*Cd133*	N/D	N/D	N/D
*Sox2*	↑	↑	↑
*Cdkn2a*	↑	↑	↑
*CyclinD*	↑	↑	↑
*Trp53*	↑	↑	↑
*Pten*	N/D	↓	↓
*Hif1a*	N/D	N/D	N/D
*Vegfa*	N/D	↓	↓
*Pdgfra*	N/D	N/D	↑
*Mgmt*	↓	↓	N/D
*Abcb1*	N/D	N/D	↑
Protein content			
CD133	N/D	↓	N/D
HIF1a	N/D	N/D	**↑**

+—small number of mitoses per a field of view; ++—moderate numbers of mitoses per a field of view and necrosis areas; +++—numbers of mitoses per a field of view and necrosis areas; ↓—decreased relative to control; ↑—increased relative to control; N/D—no difference relative to control; *Cdkn2a*—cyclin-dependent kinase inhibitor 2A; *Pten*—phosphatase and tensin homolog; *Hif1a*—Hypoxia-inducible factor 1 alpha; *Vegfa*—Vascular Endothelial Growth Factor A; *Pdgfra*—Platelet Derived Growth Factor Receptor Alpha, *Mgmt*—O6-methylguanine-DNA methyltransferase; *Abcb1*—ATP binding cassette subfamily B member 1.

## Data Availability

All data analyzed during this study are included in this article and available from the corresponding author on reasonable request.

## Abbreviations

ABCB1	ATP binding cassette subfamily B member 1
CDK	cyclin-dependent kinases
CDKN2A	cyclin-dependent kinase inhibitor 2A
CSCs	cancer stem cells
DMBA	7,12-Dimethylbenz[a]anthracene
EMT	epithelial-to-mesenchymal transition
ENU	N-ethyl-N-nitrosourea
GAPDH	glyceraldehyde 3-phosphate dehydrogenase
GBM	glioblastoma
HIF-1α	Hypoxia-inducible factor 1 alpha
IDH	Isocitrate dehydrogenase
MDM2	Mouse double minute 2 homolog
MGMT	O6-methylguanine-DNA methyltransferase
PDGFRα	Platelet Derived Growth Factor Receptor Alpha
PI3K	phosphoinositide 3 kinase
PSB	protein solubilization buffer
PTEN	phosphatase and tensin homolog
PVDF	polyvinylidene difluoride
SDS-PAGE	sodium dodecyl sulfate–polyacrylamide gel electrophoresis
VEGFA	Vascular Endothelial Growth Factor A
WB	Western blotting
WHO	World Health Organization

## Appendix A

**Table A1:** Used qPCR-RT primer sequences (all selected precisely for rat species).

Primer	Forward Sequence	Reverse Sequence
*CD133*	CTGGGGGATCAGGCCAACTA	AATTCAAAGCCCCGGGAGTG
*Sox2*	TCCATGGGCTCTGTGGTCAA	CATGTGCAGTCTACTGGGCG
*Cdkn2a*	GTACCCCGATACAGGTGATGAT	GATACCGCAAATACCGCACG
*CyclinD*	ACCTGTGAGGAAGCCATTCG	CCAGCGTGTCCCTTCTCATT
*Trp53*	AGGGAGTGCAAAGAGAGCAC	GTCTTCGGGTAGCTGGAGTG
*Pten*	GCCAAATTTAACTGCAGAGTTGC	GCGCCTCTGACTGGGAATAG
*Hif-1a*	TCACAGTCGGACAACCTCAC	TGCTGCAGTAACGTTCCAATTC
*Vegfa*	GCAGACAGTGCTCCAGCC	CCTGGGACCACTTGGCAT
*Pdgfra*	ATTGTGCTCCGTAGTCCCCA	TCCTAGCCAGGGATCTTGCC
*Abcb1*	GTCCACCATCCAGAACGCAG	GCACCTCAAATACTCCCAGCTC
